# Lifestyle Challenges Among Older Female Cancer Survivors

**DOI:** 10.1093/geroni/igaa057.1548

**Published:** 2020-12-16

**Authors:** Jessica Krok-Schoen, Janell Pisegna, Elizabeth Arthur, Emily Ridgway, Ashley Rosko

**Affiliations:** 1 Ohio State University, Columbus, Ohio, United States; 2 The Ohio State University, Columbus, Ohio, United States; 3 The James Cancer Hospital, The Ohio State University, Columbus, Ohio, United States

## Abstract

The American Cancer Society recommends that survivors maintain a healthy lifestyle including a normal weight, being physically active, and maintaining a healthy diet to improve prognosis and health-related quality of life (HRQoL). Unfortunately, the majority of cancer survivors do not engage in a healthy lifestyle. The largest proportion of cancer survivors are older adults (≥65 years), yet they are often understudied, particularly regarding healthy lifestyles. This study sought to examine the lifestyle behaviors (maintaining healthy weight, dietary intake, physical activity) of older female cancer survivors and to identify associations with HRQoL. Older female cancer survivors (n=170) completed surveys to assess HRQoL (RAND-36), diet quality (HEI-2015), physical activity, malnutrition, and BMI. Descriptive analyses, correlations, and stepwise linear regressions were utilized. The majority of the sample (mean age=74.67±8.43 years) were white (90%), married (54.4%), college-educated (63.9%), and breast cancer survivors (67.4%). Self-reported health was very good (42.6%) and good (39.6%) and general HRQoL was 59.48±15.34 out of 100. Self-reported physical activity was low; 75.3%, 54.2%, and 68.1% reported no strenuous, moderate, and mild physical activity, respectively. Mean BMI was 27.71±6.24 with 64% of the participants being overweight or obese. Mean HEI-2015 scores were 66.39±10.00, below the “good” diet quality score of 80. Risk of malnutrition was present in 27.4% of participants. Regressions found that being White (β=-0.528, p=0.001) and lower BMI (β =-0.405, p=0.024) were significant predictors of HRQoL. Results indicate the need for tailored health coaching for older cancer survivors regarding their lifestyle behaviors to improve prognosis and HRQoL.

